# Adaptation to Aquatic and Terrestrial Environments in *Chlorella vulgaris* (Chlorophyta)

**DOI:** 10.3389/fmicb.2020.585836

**Published:** 2020-10-15

**Authors:** Siegfried Aigner, Karin Glaser, Erwann Arc, Andreas Holzinger, Michael Schletter, Ulf Karsten, Ilse Kranner

**Affiliations:** ^1^Department of Botany, University of Innsbruck, Innsbruck, Austria; ^2^Institute of Biological Sciences, University of Rostock, Rostock, Germany

**Keywords:** adaptation, green algae, dehydration, desiccation, metabolomics, metabolite, microalgae

## Abstract

The globally distributed green microalga *Chlorella vulgaris* (Chlorophyta) colonizes aquatic and terrestrial habitats, but the molecular mechanisms underpinning survival in these two contrasting environments are far from understood. Here, we compared the authentic strain of *C. vulgaris* from an aquatic habitat with a strain from a terrestrial high alpine habitat previously determined as *Chlorella mirabilis*. Molecular phylogeny of SSU rDNA (823 bp) showed that the two strains differed by one nucleotide only. Sequencing of the ITS2 region confirmed that both strains belong to the same species, but to distinct ribotypes. Therefore, the terrestrial strain was re-assessed as *C. vulgaris*. To study the response to environmental conditions experienced on land, we assessed the effects of irradiance and temperature on growth, of temperature on photosynthesis and respiration, and of desiccation and rehydration on photosynthetic performance. In contrast to the aquatic strain, the terrestrial strain tolerated higher temperatures and light conditions, had a higher photosynthesis-to-respiration ratio at 25°C, still grew at 30°C and was able to fully recover photosynthetic performance after desiccation at 84% relative humidity. The two strains differed most in their response to the dehydration/rehydration treatment, which was further investigated by untargeted GC–MS-based metabolite profiling to gain insights into metabolic traits differentiating the two strains. The two strains differed in their allocation of carbon and nitrogen into their primary metabolites. Overall, the terrestrial strain had higher contents of readily available nitrogen-based metabolites, especially amino acids and the polyamine putrescine. Dehydration and rehydration led to differential regulation of the amino acid metabolism, the tricarboxylic acid cycle and sucrose metabolism. The data are discussed with a view to differences in phenotypic plasticity of the two strains, and we suggest that the two genetically almost identical *C. vulgaris* strains are attractive models to study mechanisms that protect from abiotic stress factors, which are more frequent in terrestrial than aquatic habitats, such as desiccation and irradiation.

## Introduction

Microalgae in the genus *Chlorella* (Trebouxiophyceae) are found in almost all geographic regions. The genus comprises species in freshwater lakes, soil, marine, brackish and terrestrial habitats, and some species are also symbionts of lichens, protozoa and invertebrates (Luo et al., 2010; Bock et al., 2011; Darienko et al., 2015). *Chlorella* spp. are amongst the best studied phycological model organisms and are widely used in mass cultivation systems for biotechnological applications (Safi et al., 2014). Before molecular tools for determining phylogenetic relationships became available, a large number of morphologically similar, asexually reproducing, coccoid green microalgae were described as *Chlorella* spp. The phylogeny of this genus was frequently revised, including changes in the morphological classification criteria, now covering species with mucilaginous envelopes, colony-forming species and species with bristle and spine formation (Luo et al., 2010; Bock et al., 2011).

When Beijerinck (1890) first described the authentic *Chlorella vulgaris* strain (SAG 211-11b) from an aquatic habitat, he already expected that specific environmental conditions can affect “the characteristics of the organisms.” The occurrence of aquatic and terrestrial strains makes *Chlorella* spp. promising taxa to study mechanisms of phenotypic plasticity and adaptation (Darienko et al., 2019). Compared to their aquatic counterparts, terrestrial algae are exposed to greater variations in temperature, higher irradiation and a desiccating atmosphere. Mechanisms that protect terrestrial chlorophytes from these environmental factors include photoprotectants and osmoprotectants (Gustavs et al., 2010, 2011), and/or self-shading through the formation of cell aggregates (e.g., *Apatococcus* spp.), cell colonies (e.g., *Coccomyxa* spp.), multi-layered filaments (e.g., *Klebsormidium* spp.) and the excretion of extracellular mucilage, which may contribute to a protective matrix (e.g., in biological soil crusts; Karsten and Holzinger, 2014). Species from high alpine habitats are exposed to particularly challenging environmental conditions, including extreme diurnal temperature fluctuations with freeze-thaw cycles even in the summer, high irradiation including ultraviolet radiation (UVR) and frequent winds fostering a desiccating atmosphere (Karsten and Holzinger, 2014, and references therein).

The objective of the present study was to compare the authentic, aquatic *C. vulgaris* strain with a strain from a terrestrial, high alpine habitat previously identified as *Chlorella* cf. *mirabilis* (Brunner, 2012; strain ASIB BB67). Molecular phylogenetic analysis of the SSU sequences and ITS2 secondary structure revealed a very close relationship between the two strains, which led us to consider the alpine strain as *C. vulgaris*, as described in detail below. We studied the effects of irradiance and temperature on growth, and the effects of temperature on photosynthesis and respiration, and the effects of desiccation and rehydration on photosynthetic performance. In addition, untargeted GC–MS-based metabolite profiling was used to gain insights into metabolic traits differentiating both *C. vulgaris* strains. The results are intended to contribute new data on the ecophysiology and biochemistry of these two *C. vulgaris* strains, also providing insights into the phenotypic plasticity of *C. vulgaris*.

## Materials and Methods

### Algal Strains, Culture Conditions and Microscopy

The algal strain *C. vulgaris* (SAG 211-11b) was obtained from the Culture Collection of Algae at Göttingen University, Germany (SAG), initially collected and isolated from a pond near Delft, Netherlands at 0 m a.s.l. in 1889 by Beijerinck. We chose to use the authentic strain, because it is well-described and because the culture conditions applied are known to allow only asexual reproduction (autospore formation), thus eliminating meiotic recombination, albeit we cannot exclude genomic changes due to mutations over time (Lakeman et al., 2009). However, no genomic differences were detected in duplicate strains of the same isolates of *C. vulgaris* SAG 211-11b maintained in several different culture collections for up to 50 years under different environmental conditions and transfer regimes (Müller et al., 2005). This might be explained by the extremely low reproduction rates under low light and nutrient conditions in stock culture. The high alpine terrestrial strain, previously determined as *C.* cf. *mirabilis* (ASIB BB67), was taken from the culture collection of algae of the Department of Botany, University of Innsbruck (ASIB), initially collected and isolated from soil, Liebener Rippe, Obergurgl, Austria, at 2710 m a.s.l., in 2009 by Brunner and Gärtner.

Algae were cultured in liquid Bolds Basal Medium plus vitamins (BBM + V) under a day-night cycle (16 h light at 20 to 25 μmol photons m^–2^ s^–1^ at 20°C and 8 h dark at 15°C). Cultures in the exponential growth phase were used in all experiments. Cultures were investigated by a Zeiss Axiovert 200 M microscope, equipped with a 63 × (1.4 numerical aperture) objective lens and images were generated by differential interference contrast (DIC) and captured with an Axiocam MRc5 camera controlled by Zeiss Axiovision software. For determination of cell dimensions, a minimum of 20 cells were measured.

### Molecular Phylogeny

Genomic DNA of the high alpine strain determined as *C.* cf. *mirabilis* (ASIB BB67) was extracted using the DNeasy Plant Mini Kit (Qiagen GmbH, Hilden, Germany) according to the manufacturer’s instructions. The nucleotide sequence of the SSU rDNA and ITS2 was amplified using Taq PCR Master Mix Kit (Bioline) and the primers Eaf3 and ITS055r (Marin et al., 2003). The sequence obtained for ASIB BB67 was compared with available sequences from the *Chlorella* and *Prasiola* clades (Darienko et al., 2010, 2016; Hodač et al., 2016). Multiple alignments were generated by muscle alignment implemented in MEGA (version 6.0; Tamura et al., 2013). A phylogenetic tree was constructed in MrBayes 3.2.2 (Ronquist and Huelsenbeck, 2003), using the evolutionary model GTR + G + I, with 5,000,000 generations. The reliability of tree topology was verified by maximum-likelihood analysis (GTR + I + G) using the program GARLI 2.0 (Zwickl, 2006) with 1,000 bootstrap replicates. Sequences were deposited in GenBank under the accession numbers MK397079 and MT108181.

For determination of secondary structure, the ITS2 regions were aligned and helices identified according to previously published secondary structures (Glaser et al., 2017, and references therein). The helices were folded with the online software mfold (Zuker, 2003); for visualization the online tool PseudoViewer (Byun and Han, 2009) was used.

### Light and Temperature Treatments

Growth rates in response to different photon fluence densities (PFDs) and different temperatures were monitored as the increase in chlorophyll *a* fluorescence over time as an indicator of biomass accumulation (Gustavs et al., 2009). Aliquots of 20 μL per well of liquid algal cultures containing 1–2 mg chlorophyll *a* L^–1^ grown for 5 days at 15°C were transferred to 24-well microplates (Costar, Corning GmbH, Kaiserslautern, Germany) containing 980 μL BBM + V per well. The effects of various PFDs were tested in temperature-controlled growth cabinets at 15°C for five different light regimes (8, 15, 30, 70, and 105 μmol photons m^–2^ s^–1^) under a light/dark cycle of 16:8 h L/D. Temperature-dependent growth was measured at six different temperatures (5, 10, 15, 20, 25, and 30°C) at 20 to 25 μmol photons m^–2^ s^–1^ and a light/dark cycle (16:8 h), using temperature-controlled growth chambers and a purpose-built algal incubator (Pfaff et al., 2016).

Chlorophyll *a* fluorescence was measured with a SpectraMax M2e multiplate reader (MPR; Molecular Devices, Biberach, Germany) after Donner et al. (2017). Briefly, relative fluorescence units (RFUs) were measured (λ_Ex_: 480 nm, λ_Em_: 680 nm, top read) every 24 h for 10 days, with four (light treatment) or eight (temperature treatment) replicates for each strain. Before applying the various light- and temperature regimes, cultures were pre-treated for 4 days with 25 μmol photons m^–2^ s^–1^ and at 20°C. Irradiation measurements were carried out with a Li-Cor LI-190SA cosine-corrected sensor connected to a Li-250 light meter (LI-COR Biosciences, Lincoln, NE, United States) and target PFDs were adjusted by using neutral gray plastic filter foil (Lichttechnik Hahne, Düsseldorf, Germany). The relative growth rate per day (μ d^–1^) of the two *Chlorella* strains was calculated as RFU at each time interval (*F*_t_: fluorescence after t days) using the equation

$$\mu =\ln\left(F_{t}/F_{0}\right)/t$$

where *F*_0_ is the initial fluorescence and μ d^–1^ the relative growth rate using the fitting model of Pfaff et al. (2016). The advantages of chlorophyll *a* fluorescence measurements as proxy for growth are described in detail in Gustavs et al. (2009).

### Temperature Requirements for Photosynthesis and Respiration

Oxygen evolution rates at temperatures from 5 to 50°C were measured with a Fibox 3 oxygen optode (Presens, Regensburg, Germany) using a 3 mL thermostatic transparent acrylic chamber (DW1, Hansatech, Norfolk, United Kingdom) after Remias et al. (2010). Algal suspensions were adjusted to optical densities (OD_680_) between 0.22 and 0.23 with BBM + V and enriched with 0.2 mL NaHCO_3_ solution (75 mM; Merck, Darmstadt, Germany) to produce a 3 mL suspension with a final inorganic carbon concentration of 5 mM. The PFD was adjusted to 200 μmol photons m^–2^ s^–1^ PAR emitted from a halogen light source and calibrated inside the chamber using a radiometer (QRT1 sensor, Hansatech, Norfolk, United Kingdom). Temperature was controlled using a water bath (K20/DC 10, Thermo Haake, Karlsruhe, Germany) connected to the chamber. Oxygen evolution rates (defined as the difference between oxygen production and oxygen consumption rates) were measured for 10 min each in the light, and in the dark after a 20 min pre-acclimation phase for each temperature (5 to 50°C in 5°C steps). Oxygen evolution rates for each temperature and time interval were normalized to the concentration of total chlorophyll *a*, analyzed and calculated after Porra et al. (1989). To measure chlorophyll *a*, the 3 mL algal suspension was filtered onto a Whatman GF/F glass fiber filter (No. 28418444), which was immediately frozen in liquid nitrogen and freeze-dried (Lyovac GT2, Leybold, Köln, Germany). Freeze-dried algae on filters were ground with a Mikro-Dismembrator (Sartorius, Göttingen, Germany) and extracted with 1 mL N,N-dimethylformamide (DMF, Scharlau, Sentmenat, Spain) at −20°C.

### Effects of Dehydration and Rehydration on Photosynthesis

Algal suspensions (100 μL; OD_680_ between 0.15 and 0.2) from cultures in the exponential growth phase were transferred to Whatman GF/F glass fiber filters (8 mm, Whatman, Dassel, Germany) and grown for 5 days on solid BBM + V (1.25% agar) as described under “culture conditions” above. 0.85 ± 0.2 mg (DW basis) of algae placed on filters were supplied with 20 μL BBM + V liquid medium before they were placed in a desiccation chamber (modified after Karsten et al., 2014) at 20 μmol photons m^–2^ s^–1^ at 22°C ± 1°C for 180 min above 100 ml of saturated KCl solution, resulting in a relative air humidity (RH) of 84% inside the chamber, recorded with a data logger (PCE-MSR145S-TH, PCE Instruments, Meschede, Germany). The effective quantum yield of photosystem (PS)II (YII) was measured through the transparent lid of the chamber using a pulse-amplitude modulated (PAM) fluorimeter (PAM 2500, Heinz Walz GmbH, Effeltrich, Germany), while the distance between the light probe and algal material was kept constant (6 mm). In addition, electron transport rates (ETRs) were measured before dehydration (controls) and at the end of the rehydration experiment. Algae were 30 min dark-adapted on filters on agar plates and exposed to 16 PFDs from 0 to 1660 μmol photons m^–2^ s^–1^ (for 30 s each) according to Herburger et al. (2015). The ETR values were calculated according to Schreiber and Bilger (1993) and photosynthesis-irradiance (PI) curve data were fitted according to Walsby (1997) or Webb et al. (1974), respectively, depending on whether photoinhibition occurred or not, and the following parameters calculated: the linear curve increase at limiting PFDs (α), slope of photoinhibition at high PFDs (β), the maximum electron transport rate (ETR_max_) and the initial value of light-saturated photosynthesis (I_k_; μmol photons m^–2^ s^–1^).

### GC-MS-Based Metabolite Profiling

Cultures were filtered onto Whatman GF/F glass fiber filters (No. 28418441), immediately frozen in liquid nitrogen and freeze-dried (Lyovac GT2, Leybold, Köln, Germany). Freeze-dried material was ground with glass beads using a laboratory mill (Tissuelyser II, Qiagen, Venlo, Netherlands) at 30 Hz for 3 min and resuspended in the required solvent.

Metabolite profiling was carried out using the slightly modified method of Fiehn (2016) as previously described (Gerna et al., 2018; Rippin et al., 2019; Arc et al., 2020) after optimization using test samples. Briefly, aliquots of freeze dried and finely ground material and quality controls, including commercially available standards and blanks, were extracted at −20°C in water: acetonitrile: isopropanol (2:3:3) containing ^13^C_6_-sorbitol and ^13^C_5_, ^15^N-valine as internal standards. Insoluble material was pelleted by centrifugation and an aliquot of the supernatant was collected and dried in a vacuum centrifuge. Thereafter, metabolites were derivatized by successive incubations with methoxyamine in pyridine solution and N-methyl-N-trimethylsilyl-trifluoroacetamide (MSTFA). Metabolites were separated on a Rxi-5SilMS column (Restek, 30 m with a 10 m Integra-Guard column) using a Trace 1300 gas chromatograph in splitless mode and detected by a TSQ8000 triple quadrupole mass spectrometer (Thermo Scientific, Waltham, MA, United States). A mix of alkanes was injected in the middle of the queue for external retention index calibration, in addition to quality controls. The “Automated Mass-spectral Deconvolution and Identification System” (AMDIS) was used to extract compound spectra from the raw data files and to compare them against a custom-built mass spectral library and commercial or publicly available databases, including the NIST, Golm and Fiehn databases (Kopka et al., 2005; Kind et al., 2009). Peak areas for compound-specific trace ions were determined using the Xcalibur software (Thermo Scientific) allowing for relative quantification of identified and unknown compounds from the different samples.

### Analysis of Photosynthetic Pigments

For analysis of photosynthetic pigments, the freeze-dried and finely ground powder (as described above for metabolite profiling) was suspended in 1.5 mL acetone (MTBE, SigmaAldrich, St. Louis, MO, United States) containing 0.01% butylated hydroxytoluene (BHT) (SigmaAldrich, St. Louis, MO, United States) and analyzed after Remias et al. (2010) with minor modifications. Extracts were shaken on a orbital shaker (Thermo Scientific Compact Digital Microplate Shaker) for 10 min at 1200 rpm and 4°C, and the supernatant was removed, evaporated to dryness in a SpeedVac (SPD111V, Thermo Fisher Scientific, Waltham, MA, United States) and re-suspended in 250 μL N,N-DMF, followed by centrifugation (15,000 × *g*, 45 min, 4°C) prior to injection into the HPLC. Pigments were separated by HPLC (1100, Agilent Technologies, Waldbronn, Germany) using a LiChroCART column (C18, 100 × 4.6 mm, 5 μm, 120 Å) at a flow rate of 1 mL min^–1^ and detected using a diode array detector (DAD) (Agilent Technologies, Waldbronn, Germany) set to 440 nm for carotenoids and 662 nm for chlorophylls. Solvent A was acetonitrile:methanol (74:6) and solvent B was methanol:hexane (5:1). Separation was started at 0% solvent B for 4 min, followed by a gradient to 100% solvent B from 4 to 9 min, which was maintained for 9 min, followed by a 5 min post-run with 0% solvent B. All solvents were of HPLC-grade quality. Chlorophyll *a* was obtained from SigmaAldrich, St. Louis, MO, United States; antheraxanthin and violaxanthin from DHI C14, Centralen, Denmark; zeaxanthin and lutein from Carl Roth, Karlsruhe, Germany; β-carotene from Calbiochem, Darmstadt, Germany. Neoxanthin and chlorophyll *b* from spinach extracts were collected with a fraction collector (1200 Series, Agilent, Waldbronn, Germany) and concentrations calculated using the specific absorption coefficients.

### Statistical Analysis

Statistical evaluation of the data was performed with R (R Core Team, 2020) using the ellipse, lsmeans (Lenth, 2016) and ggplot2 packages (Wickham, 2016). Physiological data were tested for significance by two-way ANOVA, followed by Tukey’s multiple comparison test and subgroups with significantly different means were identified at *P* < 0.05. Metabolites were reported as differentially accumulated when the false discovery rate (FDR; Benjamini and Hochberg, 1995) corrected two-way ANOVA *P* value was below 0.01 with a log_2_ ratio higher than 1. Details of statistical treatments are shown in Supplementary Table S1.

## Results

### Morphology and Molecular Phylogeny of the Investigated Strains

Mature cells of both strains were spherical, sometime oval, and those of the authentic *C. vulgaris* SAG 211-11b strain were significantly smaller (5.47 ± 0.44 versus 6.57 ± 0.66; *n* = 20, mean value ± SD; *P* < 0.05; Supplementary Figure S1) than those of the high alpine *Chlorella* cf. *mirabilis* ASIB BB67 strain (in this study re-assessed as *C. vulgaris*, see below).

**FIGURE 1:**
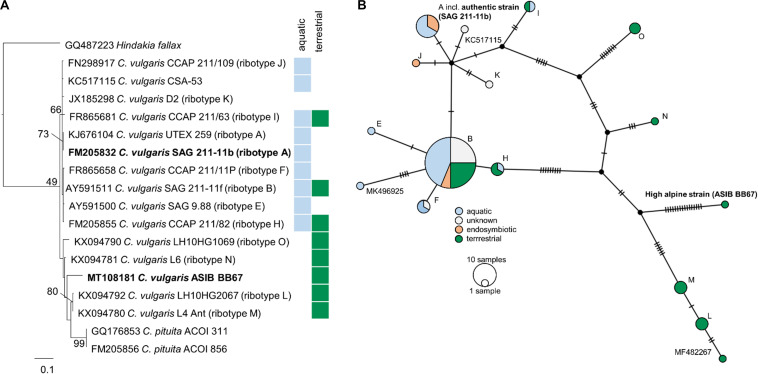
Molecular phylogeny and ribotype network of *Chlorella vulgaris*. **(A)** Molecular phylogeny of ITS2 sequence comparisons of *Chlorella vulgaris*. **(B)** Ribotype network (TCS calculated by PopArt) of 83 *Chlorella vulgaris* ITS sequences. Each hatch marks one mutation. Ribotype assignment was conducted according to Hodač et al. (2016); sequences not yet assigned to a ribotype are indicated by their accession numbers. Colors indicate habitats (blue: aquatic; green: terrestrial) or symbiotic life-style (orange) of the strains.

Phylogenetic analyses of the SSU rDNA (823 bp) of both strains confirmed their positions in the *Chlorella* clade of the Trebouxiophyceae and revealed a very close relationship between the high alpine *C.* cf. *mirabilis* strain (ASIB BB67) and the authentic *C. vulgaris* (SAG 211-11b) differing in only one base pair (Supplementary Figure S2). Therefore, the high alpine *C.* cf. *mirabilis* strain (ASIB BB67) was considered to be *C. vulgaris*. As the high alpine strain was isolated from soil, it is hereafter referred to as “terrestrial *C. vulgaris*.” The authentic strain was isolated from an aquatic habitat and is referred to as “aquatic *C. vulgaris*.” Although the two *C. vulgaris* strains differed by 26 nucleotides in the ITS2 region, no compensatory base changes were detected. Sequencing of the ITS2 region confirmed that both strains belong to the same species, but the terrestrial *C. vulgaris* belongs to another ribotype than the aquatic one. Importantly, compared to aquatic and pre-dominantly aquatic strains, ribotypes from terrestrial strains showed considerably more mutations in the ITS2 regions (Figure 1).

### Effects of Light and Temperature on Growth Rates, and Temperature Dependence of Photosynthesis and Respiration

**FIGURE 2:**
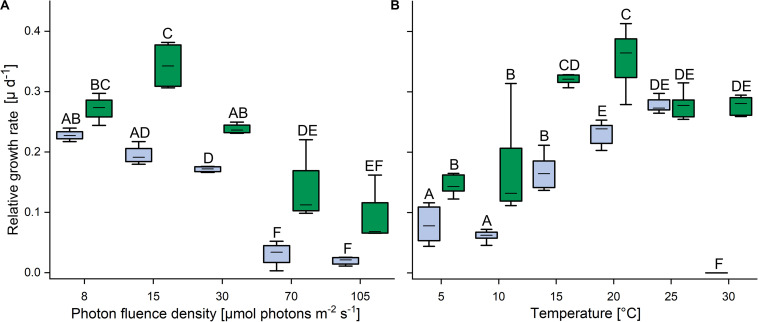
Effects of light and temperature on the growth of two *Chlorella vulgaris* strains. **(A)** Dependence of growth rate on photon fluence density (*n* = 4, mean value ± SD) and **(B)** on temperature (*n* = 8, mean value ± SD). Blue color denotes the aquatic strain and green color shows the terrestrial strain. Capital letters in either panels **(A)** or **(B)** indicate significant differences assessed by two-way ANOVA followed by Tukey’s *post hoc* test (*P* < 0.05).

In response to increasing PFDs, the terrestrial strain showed higher growth rates than the aquatic strain (Figure 2A). At 8 μmol photons m^–2^ s^–1^, the growth rates of the aquatic *C. vulgaris* were highest and fell with increasing PFDs, and at 70 and 105 μmol photons m^–2^ s^–1^ almost no growth was detected. By contrast, the terrestrial *C. vulgaris* showed highest growth rates at 15 μmol photons m^–2^ s^–1^, which also declined at higher PFDs (Figure 2A). In response to increasing temperature, growth rates increased with temperature in both strains between 10 and 20°C (Figure 2B). The terrestrial strain grew significantly better than the aquatic one between 5 and 20°C, with a maximum at 20°C, and at 25°C the growth rates of both strains converged. However, at 30°C the aquatic strain did not grow anymore, whereas the terrestrial strain still grew well (Figure 2B).

**FIGURE 3:**
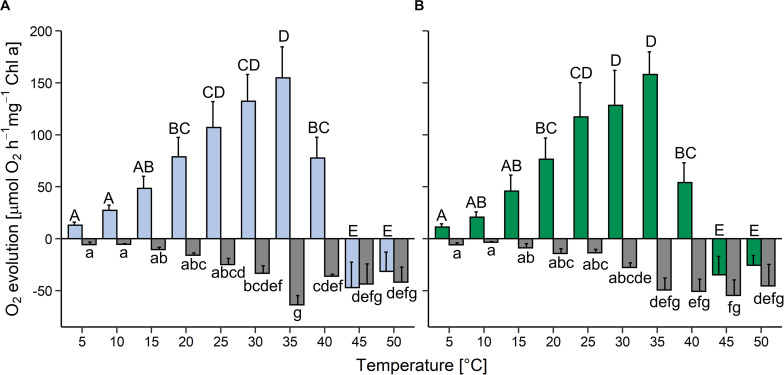
Effects of temperature on photosynthesis and respiration in two *Chlorella vulgaris* strains. Oxygen evolution in the light at 200 μmol photons m^–2^ s^–1^ (colored bars) and oxygen consumption in the dark (gray bars) is shown in panel **(A)** for the aquatic strain (blue and gray) and **(B)** for the terrestrial strain (green and gray). Data show means ± SD (*n* = 4). Different capital letters (for oxygen release in the light) and small letters (for oxygen consumption in the dark) indicate significant differences calculated by two-way ANOVA followed by Tukey’s *post hoc* test (*P* < 0.05).

In the light, oxygen production (i.e., oxygen production by photosynthesis minus oxygen consumption by respiration and other oxygen-consuming processes) increased between 5 and 35°C in both strains and then declined rapidly; above 45°C oxygen consumption was higher than oxygen release (colored bars in Figure 3). Compared to the maximum values at 35°C, at 40°C gross oxygen production was reduced by approximately 50% in the aquatic strain and by about 70% in the terrestrial strain and then ceased at 45°C. In both strains, oxygen consumption in the dark (i.e., by respiration and other oxygen-consuming processes) was very low at 5 and 10°C, then gradually increased up to a temperature of 35°C (gray bars in Figure 3). Between 40 and 50°C oxygen consumption plateaued in the aquatic strain, whereas it plateaued between 35 and 50°C in the terrestrial strain. At 25°C, the terrestrial strain showed a two-fold higher photosynthesis:respiration (P:R) ratio than the aquatic strain (Supplementary Figure S3).

### Effects of Dehydration and Rehydration on Photosynthesis

**FIGURE 4:**
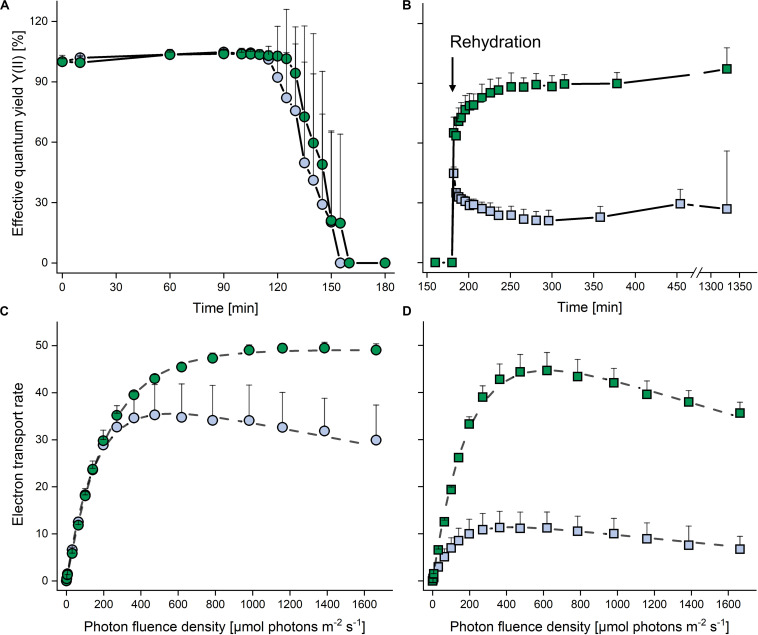
Effects of dehydration and rehydration on photosynthesis in two *Chlorella vulgaris* strains. Blue symbols denote the aquatic strain and green symbols show the terrestrial strain. **(A)** Effective quantum yield during dehydration at 84% relative humidity and **(B)** during the subsequent rehydration at 95% relative humidity; data are means ± SD (*n* = 5) expressed as a percentage of the initial values at time 0. **(C)** Electron transport rates of algae before dehydration (controls) and **(D)** after the end of rehydration; data are means ± SD (*n* = 4). Two-way ANOVA (*P* < 0.05) revealed significant differences between the two strains in panels **(B–D)**.

In both strains, the YII remained at >0.6 (set to 100% in Figure 4A) for 120 min after exposure to desiccating conditions above a saturated KCl solution (corresponding to a RH of 84%), and then decreased to zero within the next 30 min. The cultures were left for another 30 min in this condition, and upon subsequent rehydration at ∼95% RH (Figure 4B), the terrestrial strain recovered rapidly, regaining 80% of the YII values of non-dehydrated cells after 20 min, and then further increased back to control levels at the end of the rehydration phase. By contrast, the aquatic strain recovered only about one third of the YII values of non-dehydrated cells.

In non-dehydrated controls, ETRs increased up to 1600 μmol photons m^–2^ s^–1^ in the terrestrial strain (α: 0.224 ± 0.002, I_k_: 218.7 ± 5.7; *P* < 0.05), whereas the aquatic strain (α: 0.270 ± 0.005, I_k_: 151.7 ± 26.7; *P* < 0.05) showed photoinhibition (β = −0.007 ± 0.001) at PFDs above 500 μmol photons m^–2^ s^–1^ (Figure 4C), with a significantly higher (*P* < 0.05) ETR_max_ value for the terrestrial strain. At the end of the rehydration experiment, the terrestrial strain revealed photoinhibition (β = −0.017 ± 0.005; Figure 4D), although ETR_max_ and I_k_ did not differ significantly from the values of non-dehydrated controls (Figure 4C). However, in the aquatic strain, the ETR curve had a significantly lower α value (0.106 ± 0.035; *P* < 0.05) and the ETR_max_ value recovered to only about 30% when compared to the non-desiccated cells, whereas the I_k_ (130.4 ± 9.9) was not significantly reduced (Figure 4D).

### Effects of Dehydration and Rehydration on Metabolite Composition

**FIGURE 5:**
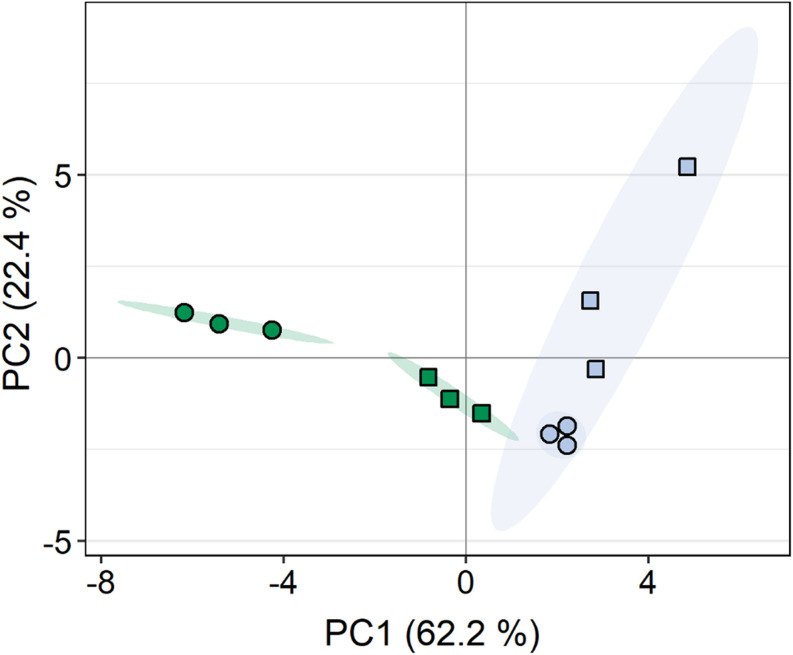
Principal component analysis of metabolite profiles in response to dehydration and rehydration of two *Chlorella vulgaris* strains. Blue symbols denote the aquatic strain and green symbols show the terrestrial strain. Circles show algae before dehydration (controls) and rectangles show algae after the end of rehydration (*n* = 3).

A total of 108 compounds were detected by GC-MS-based metabolite profiling, of which 94 metabolites were identified. Principal component analysis (PCA) revealed clear differences in the metabolite profiles of both *C. vulgaris* strains along principal component (PC) 1 and PC2, respectively, accounting for 62.2 and 22.4% of the total variance (Figure 5). The dehydration/rehydration treatment led to clear changes in the metabolite profile of the terrestrial *C. vulgaris*, resulting in two distinct clusters on the PCA plot, whereas the clustering according to the dehydration/rehydration treatment for the aquatic *C. vulgaris* was less pronounced.

**FIGURE 6:**
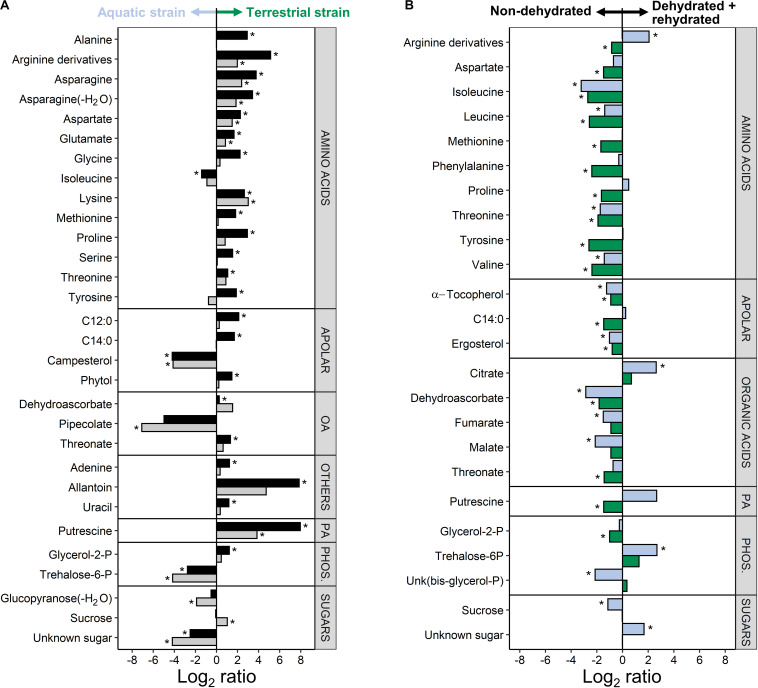
Difference in the metabolite profiles of the two *Chlorella vulgaris* strains, and in response to dehydration and rehydration. **(A)** Differences in metabolites between the two strains before dehydration (black bars) and after the end of rehydration (gray bars) shown as log_2_ ratios (metabolite contents in the terrestrial strain divided by the metabolite contents in the aquatic strain). A positive log_2_ ratio indicates that a given metabolite has a higher content in the terrestrial strain and a negative log_2_ ratio indicates a higher content in the aquatic strain (see arrows on top of the panel). **(B)** Effects of dehydration followed by rehydration (D + R) on the metabolites of the aquatic strain (blue bars) and of the terrestrial strain (green bars) shown as log_2_ ratios (metabolite contents after D + R divided by the metabolite contents in non-dehydrated controls). A positive log_2_ ratio indicates that a given metabolite has a higher content after D + R and a negative log_2_ ratio indicates a lower content after D + R (see arrows on top of the panel). Differences are only shown for the most prominent differences (FDR corrected two-way ANOVA at *P* < 0.01; Log_2_ ratios >1). Asterisks indicate significant differences in metabolite contents between the aquatic and the terrestrial strain in panel **(A)** and between the non-dehydrated controls and samples subjected to the D + R treatment in panel **(B)** (FDR corrected *P* < 0.05).

A comparison of both *C. vulgaris* strains showed that 30 metabolites were differently accumulated across treatments, i.e., non-dehydrated controls and samples measured at the end of rehydration (FDR corrected two-way ANOVA, *P* < 0.01, log_2_ ratios >1; Figure 6A and Supplementary Table S1). Eleven amino acids, three organic acids, two free fatty acids and other compounds such as putrescine and allantoin were up-accumulated in the terrestrial strain. Six compounds were up-accumulated in the aquatic strain, especially pipecolate, campesterol, and trehalose-6-phosphate (Figure 6A).

A comparison of the two treatments showed that 24 metabolites were differently accumulated across both strains (FDR corrected two-way ANOVA, *P* < 0.01, log_2_ ratios >1; Figure 6B and Supplementary Table S1), whereby the two *C. vulgaris* responded differently to the treatments. The dehydration/rehydration treatment lead to a down-accumulation of 9 amino acids, with the notable exception of Arg derivatives, which were up-accumulated in the aquatic strain. Dehydroascorbic acid, threonic acid, α-tocopherol, ergosterol and the tricarboxylic acid cycle (TCA) cycle intermediates fumaric and malic acid were also down-accumulated, whereas the aquatic strain accumulated citric acid and trehalose-6-phosphate. Both strains showed a different response to the dehydration/rehydration treatment regarding the accumulation of metabolites such as Arg, Pro, myristic acid and putrescine, which were all down-accumulated in the terrestrial but not in the aquatic strain. Interactive effects between the treatments and *C. vulgaris* strains were found for 26 metabolites (Supplementary Table S1), further supporting that the two strains responded differently to the treatment. Both *C. vulgaris* strains contained the photosynthetic pigments typical of the Viridiplantae, with no significant differences (Supplementary Table S2).

## Discussion

Chlorellaceae thrive in the terrestrial habitats of polar and temperate zones as well as in marine and freshwater habitats (Hodač et al., 2016; Darienko et al., 2019). We re-assessed a *C.* cf. *mirabilis* strain, isolated from a high-alpine terrestrial habitat, as *C. vulgaris*, and compared its physiology and metabolite composition to the authentic, aquatic *C. vulgaris* strain. The aquatic strain had a smaller cell diameter, but was otherwise morphologically indistinguishable from the terrestrial strain. However, the growth characteristics and temperature requirements of the terrestrial and the aquatic strain differed significantly. After exposure to desiccating conditions, the terrestrial strain recovered photosynthetic performance upon rehydration, whereas that of the aquatic strain was severely impaired, and the differences between both strains were also reflected by their metabolite profiles and metabolite re-arrangement associated to the dehydration/rehydration treatment.

### The Terrestrial and the Aquatic Strain Belong to Different Ribotypes

Molecular analysis of the SSU rDNA revealed that the aquatic and the terrestrial *C. vulgaris* strains belong to the same species, differing by one base pair only, with ≥99.5% SSU sequence similarity (Supplementary Figure S2). Such high sequence similarities are not uncommon in algae, including in polar and temperate terrestrial *C. vulgaris* strains (Hodač et al., 2016). However, the two *C. vulgaris* strains differed by 26 nucleotides in the ITS2 region, and the terrestrial strain belonged to another ribotype than the aquatic one. Hodač et al. (2016) showed 13 unique ribotypes for “true” *C. vulgaris*, differing by up to 18 nucleotides in the ITS2 region. The terrestrial strain clustered with terrestrial polar and temperate *C. vulgaris* strains (ribotypes M, N, L, and O). Ribotypes from terrestrial strains showed considerably more mutations in the ITS2 regions (Figure 1), indicative of an early separation of terrestrial from aquatic strains and their exposure to environmental conditions such as irradiation and desiccation that favor mutagenesis.

### The Terrestrial Strain Thrives Better at Increasing Irradiance and Temperature, and Tolerates Desiccation

The ability to grow under increasing irradiation and temperature was higher in the terrestrial than the aquatic strain (Figure 2). Whereas growth ceased at 70 μmol photons m^–2^ s^–1^ in the aquatic strain, the terrestrial strain still showed 30% of its maximum growth rates between 70 and 105 μmol photons m^–2^ s^–1^, comparable to the ability of polar and temperate soil algae to grow at such irradiances (Gustavs et al., 2010; Shukla et al., 2013). Furthermore, the terrestrial strain was able to grow at a wider temperature range than the aquatic strain, up to 30°C, similar as reported for *Chlorella sorokiniana* and *Chlorella ohadii*, which can grow at temperatures above 25°C (Morita et al., 2000; de-Bashan et al., 2008; Treves et al., 2017). The ability to grow over a broad temperature range may reflect the environmental conditions in high alpine habitats, which are characterized by strongly fluctuating soil temperatures from −15°C in the winter (without snow cover) and up to 40°C in summer (Körner et al., 2003). Both strains were capable of gross oxygen production from 5 to 35°C, which peaked at 35°C and ceased at 45°C (Figure 3). The P:R ratios tended to be higher in the terrestrial than the aquatic strain, with a significantly higher P:R ratio at 25°C due to a significantly lower oxygen consumption, and at 40°C, both strains still had positive P:R ratios (Figure 3 and Supplementary Figure S3). The better performance of the terrestrial strain at elevated temperatures is in agreement with an adaptation of photosynthetic and respiratory processes to higher temperatures and irradiation experienced by soil crusts (Karsten and Holzinger, 2012) compared to algae living in aquatic environments. In summary, the terrestrial strain tolerates more light and higher temperatures than the aquatic one, indicative of differences in phenotypic plasticity of the two strains.

We further investigated the response of the two strains to desiccation, and found substantial differences in their relative desiccation tolerance. Desiccation-tolerance can be defined as the ability to revive from the air-dried state, which would include the relatively mild desiccation by exposure to 84% RH used here. By contrast, desiccation tolerance *sensu stricto* is often defined as the capability to survive drying to the air-dry state at RHs below 65%, corresponding to a drop in absolute water content to or below 0.1 g H_2_O g^–1^ dry mass and a water potential of ≤−100 MPa (Vertucci and Farrant, 1995; Walters et al., 2005). Such extreme tolerance of desiccation is found in prokaryotes, algae, bryophytes, lichens and occasionally in pteridophytes, but rarely in the vegetative tissues of angiosperms. The mechanisms of desiccation tolerance are still not fully understood, although a plethora of papers is available reporting on the constitutive and inducible adaptations required to withstand the (bio)chemical, metabolic and morphological modifications occurring upon desiccation (for reviews on resurrection plants, mosses and lichens see Proctor et al., 2007; Kranner et al., 2008; Farrant and Moore, 2011, respectively). Of the desiccation tolerant life-forms investigated, green algae belong to the least studied organisms (for review see Karsten and Holzinger, 2014), and with the exception of lichenized algae, it is not even clear which free-living green algae are desiccation tolerant *sensu stricto*.

We showed that exposure to 84% RH decreased YII, the effective quantum yield of PSII, to zero in both strains (Figure 4A). The terrestrial strain was able to recover fully upon rehydration, whereas YII was severely impaired in the aquatic strain, reaching only one third of the pre-desiccation level (Figure 4). This incomplete recovery of YII is in agreement with data by Gray et al. (2007), who also presented an attractive model system, including two *Chlorella* sp. strains, one of which is aquatic and the other isolated from desert soil, to study the response to dehydration/rehydration treatments. These authors showed that, depending on the length of exposure to desiccating conditions (24 h to 4 weeks), the terrestrial strain was able to recover between 50 and 80% of maximum quantum yield of PSII photochemistry (Fv/Fm) upon rehydration, whereas the aquatic strain recovered only about 30 to 40% of Fv/Fm. Prior to desiccation, the aquatic but not the terrestrial strain showed photoinhibition above 500 μmol photons m^–2^ s^–1^ (Figure 4C), and at the end of rehydration, the differences between the two strains became even more apparent when the ETR_max_ value of the terrestrial strain recovered almost fully (albeit with slight photoinhibition) and that of the aquatic strain to only about 30% (Figure 4D). Taken together, data presented here and by Gray et al. (2007) suggest that genetically almost identical strains of *C. vulgaris* from aquatic and terrestrial habitats are attractive models to study mechanisms that confer tolerance to “mild” desiccation (such as air drying at 84% RH).

Mechanisms that protect from desiccation-induced molecular damage are believed to include the constitutive expression of specific water-stress proteins termed “dehydrins” or “late embryogenesis abundant” proteins, an efficient antioxidant system and high levels of intracellular osmoprotectants such as non-reducing sugars and polyols in conjunction with inducible mechanisms that help avoid damage and/or repair damage incurred upon desiccation (Kranner et al., 2005, 2008; Kosugi et al., 2013). For example, changes in gene expression induced by desiccation and/or rehydration (Candotto Carniel et al., 2016; Banchi et al., 2018), protein synthesis and membrane galactoglycerolipid composition (Gasulla et al., 2013, 2016) were observed in the lichen photobionts *Trebouxia gelatinosa* and *Asterochloris erici*.

### Metabolic Profiling Before and After Dehydration Treatment

The metabolite profiles of the two strains clearly differed before and after the dehydration/rehydration experiment (Figures 5, 6), indicative of constitutive and inducible differences in metabolism. The comparison of the metabolite profiles of the terrestrial and the aquatic strain (Figure 6A) revealed that most amino acids were constitutively up-accumulated in the terrestrial strain. These included the Asp family of amino acids, Asp, Lys, Met, Thr, and amino acids involved in nitrogen assimilation and metabolism, i.e., Gln and Glu together with allantoin, a nitrogen-rich heterocyclic compound involved in purine metabolism, with a housekeeping role in nitrogen recycling and plant stress response (Takagi et al., 2016; Casartelli et al., 2019). Interestingly, green algae, including *Chlorella* sp., can use allantoin as a sole nitrogen source for growth (Prasad, 1983). With a high nitrogen to carbon ratio (Winter et al., 2015), Arg derivatives (including Orn, see Supplementary Table S1 for details), were among the most up-accumulated amino acids in the terrestrial strain. Arginine is required for polyamine synthesis in higher plants, whereas unicellular green algae have lost the arginine route and depend on putrescine biosynthesis from Orn (Fuell et al., 2010). Putrescine, a polyamine that has been associated with plant stress tolerance (Alet et al., 2011; Chen et al., 2018), was also strongly up-accumulated in the terrestrial strain. Compatible solutes such as putrescine and Pro, serve to stabilize macromolecules, including DNA, and membranes, can scavenge reactive oxygen species, and are thought to contribute to tolerance of desiccation and freezing (Hayat et al., 2012; Sadowsky et al., 2016; Chen et al., 2018), also conferring cryoprotection in green algae (Jackson and Seppelt, 1995). In higher plans, putrescine has been reported to protect the photosynthetic apparatus from oxidative damage (Alet et al., 2011). Furthermore, the up-accumulation of Gly and Ser together with increased levels of glycerate and glycolate (Supplementary Table S1) in the terrestrial strain points at a higher flux through the photorespiration pathway. Due to its involvement in the dissipation of excess light energy, using energy in at least three processes, the recycling of glycolate, the reincorporation of ammonia and the turnover of the reductive pentose phosphate pathway (Foyer and Noctor, 2000), photorespiration is likely more important to the terrestrial strain than the aquatic one. In summary, the terrestrial strain constitutively accumulates osmoprotectants and readily available nitrogen-based primary metabolites in conjunction with an apparent up-regulation of photorespiration, supporting growth and conferring protection from abiotic stress factors, such as desiccation and irradiation, required in terrestrial habitats.

Isoleucine was the only amino acid up-accumulated in the aquatic strain (Figure 6A), while Leu and Val were not differentially accumulated between the two strains (Supplementary Table S1). This suggests a differential regulation of the synthesis pathways for branched-chain amino acids (BCAAs), i.e., Leu, Val, Ile (Galili et al., 2016), compared to the syntheses of the other amino acids. Recent evidence suggests that BCAAs contribute to target of rapamycin (TOR) activation and signaling, an evolutionarily conserved hub of nutrient sensing and metabolic signaling. Of the two distinct multiprotein complexes of TOR, TORC1 is highly conserved in all eukaryotes, including algae, with established downstream processes such as protein synthesis and cell proliferation (Pérez-Pérez et al., 2017; Cao et al., 2019). Other metabolites up-accumulated in the aquatic strain included the non-proteinogenic amino acid pipecolate and trehalose-6-phosphate (Figure 6A), and trehalose was also present at higher levels in the aquatic than the terrestrial strain (Supplementary Table S1). Trehalose and pipecolate have been associated with osmoprotection, and especially trehalose is thought to be part of the mechanisms conferring desiccation tolerance. Therefore, one would intuitively expect trehalose to be up-accumulated in the terrestrial strain, and the up-accumulation in the aquatic strain was somewhat unexpected. However, the trehalose precursor trehalose-6-phosphate is receiving increasing attention due to its potential role as an important signaling metabolite, regulating carbon assimilation and sugar status in plants (Ponnu et al., 2011). In summary, the aquatic strain accumulates metabolites involved in BCAA metabolism, and it appears that the two strains differ in their allocation of carbon and nitrogen into their primary metabolites.

The metabolite profiles recorded after the dehydration/rehydration treatment showed that most amino acids were down-accumulated in the terrestrial strain, which could indicate that they were incorporated into proteins involved in desiccation tolerance or into repair mechanisms required for the progressive resumption of metabolism upon rehydration (Figure 4). Such changes were demonstrated for desiccation tolerant organisms such as bryophytes, in which dehydration led to a reduction in proteins, which returned to control values upon rehydration (Cruz de Carvalho et al., 2014). In the aquatic strain, only the BCAAs and Ile precursor Thr were down-accumulated, suggesting that the synthesis of BCAAs was impeded. By contrast, Arg derivatives and putrescine – which were constitutively high in the terrestrial strain (Figure 6A) – were up-accumulated in the aquatic strain (Figure 6B), indicative of an inducible synthesis of polyamines. Compounds involved in antioxidant defense, α-tocopherol, dehydroascorbic acid and the ascorbate catabolite Thr were down-accumulated after the dehydration/rehydration treatment in both strains, reflecting a shift in the redox environment towards more oxidizing conditions, which are generally associated with abiotic stress factors (Kranner and Birtic, 2005; Kranner et al., 2006). Furthermore, sucrose was down-accumulated in the aquatic strain, consistent with the severely compromised photosynthetic performance of the aquatic strain after the dehydration/rehydration treatment (Figure 4). In addition, the up-accumulation of citric acid and down-accumulation of fumaric and malic acid together (Figure 6B) with trends observed for other TCA cycle intermediates, aconitic acid, 2-oxoglutaric acid and succinic acid (Supplementary Table S1), indicates a regulation or disruption of the TCA cycle, conceivably through isocitrate dehydrogenase. These variations in TCA cycle intermediates were less pronounced in the terrestrial strain, consistent with its better photosynthetic performance (Figure 4). In summary, the most pronounced differences in the response of both strains to the dehydration/rehydration treatment were observed in sucrose accumulation, amino acid metabolism and TCA cycle regulation.

## Conclusion

In conclusion, in two genetically almost identical *C. vulgaris* strains, the adaptation to the terrestrial environment was associated with the ability to grow under a wider light and temperature range than the aquatic one. The two strains also differed in their degree of tolerance to mild desiccation, after which the terrestrial strain recovered photosynthetic performance fully, whereas it was severely impaired in the aquatic strain. Constitutive differences between both strains related to amino acid and polyamine metabolism, photorespiration and allocation of carbon and nitrogen within the primary metabolites appear to influence their response to the dehydration/rehydration treatment.

## Data Availability Statement

The datasets presented in this study can be found in the Supplementary Material and in online repositories. The names of the repositories and accession number(s) can be found in the article.

## Author Contributions

SA, UK, AH and IK planned and designed the ecophysiological experiments. EA, SA, and IK supervised the metabolite profiling. SA and MS conducted the ecophysiological characterization of the two algal strains, and processed the data. KG conducted DNA extraction, sequencing and molecular analysis. SA and EA conducted the GC-MS analyses. EA and IK interpreted the data. SA wrote the first draft of the manuscript, which was edited by IK, AH, KG, EA, and UK. All authors edited and approved the final version of this manuscript.

## Conflict of Interest

The authors declare that the research was conducted in the absence of any commercial or financial relationships that could be construed as a potential conflict of interest.

## Supplementary Material

Supplementary Figure 1 Light-micrographs of the two *Chlorella vulgaris* strains. **(A)** Representative cell of the authentic aquatic strain (SAG 211-11b) and panel **(B)** of the terrestrial high alpine strain (ASIB BB67) taken from cultures in the exponential growth phase; scale bars: 2 μm.

Supplementary Figure 2 Molecular phylogeny of the Trebouxiophyceae. Based on SSU rDNA sequence comparisons, inferred by Bayesian method with Bayesian Posterior Probabilities (PP) and maximum-likelihood (ML) bootstrap support (BP), nodes are indicated based on a data set of 1780 aligned positions of 47 taxa using MrBayes and GARLI. From left to right: support values correspond to ML, BP and Bayesian PP; BP values lower than 50% and PP lower than 0.8 not shown. The sister group of the *Oocystis*-lineage was chosen as outgroup. The newly sequenced strain BB67 is marked with an asterisk.

Supplementary Figure 3 Effects of temperature on photosynthesis:respiration (P:R) ratios in two *Chlorella vulgaris* strains. In panel **(A)**, blue bars show the aquatic strain and in panel **(B)** green bars show the terrestrial strain; data are means ± SD (*n* = 4). Capital letters above bars indicate significant differences assessed by two-way ANOVA followed by Tukey’s *post hoc* test (*P* < 0.05).

Supplementary Table 1 Complete metabolite profiling dataset.

Supplementary Table 2 Concentrations of photosynthetic pigments and in two *Chlorella vulgaris* strains before dehydration (controls); data are means ± SD (*n* = 3), and values did not differ statistically (*P* < 0.05).

## References

[B1] Alet A. I. Sanchez D. H. Cuevas J. C. Del Valle S. Altabella T. Tiburcio A. F. (2011). Putrescine accumulation in *Arabidopsis thaliana* transgenic lines enhances tolerance to dehydration and freezing stress. *Plant Signal. Behav.* 6 278–286. 10.4161/psb.6.2.14702 21330789 PMC3121989

[B2] Arc E. Pichrtova M. Kranner I. Holzinger A. (2020). Pre-akinete formation in *Zygnema* sp. from polar habitats is associated with metabolite re-arrangement. *J. Exp. Bot.* 71 3314–3322. 10.1093/jxb/eraa123 32147713 PMC7289716

[B3] Banchi E. Candotto Carniel F. Montagner A. Petruzzellis F. Pichler G. Giarola V. (2018). Relation between water status and desiccation-affected genes in the lichen photobiont *Trebouxia gelatinosa*. *Plant Physiol. Biochem.* 129 189–197. 10.1016/j.plaphy.2018.06.004 29894859

[B4] Beijerinck M. W. (1890). Culturversuche mit Zoochlorellen, Lichenengonidien und anderen niederen Algen. *Bot. Ztg.* 47 725–739, 741–754, 757–768, 781–785.

[B5] Benjamini Y. Hochberg Y. (1995). Controlling the false discovery rate - a practical and powerful approach to multiple testing. *J. R. Stat. Soc. B* 57 289–300. 10.1111/j.2517-6161.1995.tb02031.x

[B6] Bock C. Krienitz L. Pröschold T. (2011). Taxonomic reassessment of the genus *Chlorella* (Trebouxiophyceae) using molecular signatures (barcodes), including description of seven new species. *Fottea* 11 293–312. 10.5507/fot.2011.028

[B7] Brunner B. (2012). *Bodenalgen aus Hochalpinen Lokalitäten der Liebener Rippe (Obergurgl, Tirol)*, Master thesis, University of Innsbruck, Innsbruck, 118 pp.

[B8] Byun Y. Han K. (2009). PseudoViewer3: generating planar drawings of large-scale RNA structures with pseudoknots. *Bioinformatics* 25 1435–1437. 10.1093/bioinformatics/btp252 19369500

[B9] Candotto Carniel F. Gerdol M. Montagner A. Banchi E. De Moro G. Manfrin C. (2016). New features of desiccation tolerance in the lichen photobiont *Trebouxia gelatinosa* are revealed by a transcriptomic approach. *Plant Mol. Biol.* 91 319–339. 10.1007/s11103-016-0468-5 26992400

[B10] Cao P. Kim S. J. Xing A. Schenck C. Liu L. Jiang N. (2019). Homeostasis of branched-chain amino acids is critical for the activity of TOR signaling in *Arabidopsis*. *eLife* 8:e50747. 10.7554/eLife.50747 31808741 PMC6937141

[B11] Casartelli A. Melino V. J. Baumann U. Riboni M. Suchecki R. Jayasinghe N. S. (2019). Opposite fates of the purine metabolite allantoin under water and nitrogen limitations in bread wheat. *Plant Mol. Biol.* 99 477–497. 30721380 10.1007/s11103-019-00831-z

[B12] Chen D. Shao Q. Yin L. Younis A. Zheng B. (2018). Polyamine function in plants: metabolism, regulation on development, and roles in abiotic stress responses. *Front. Plant Sci.* 9:1945. 10.3389/fpls.2018.01945 30687350 PMC6335389

[B13] Cruz de Carvalho R. Bernardes da Silva A. Soares R. Almeida A. M. Coelho A. V. Marques (2014). Bryophyte desiccation tolerance with proteomics. *Plant Cell Environ.* 37 1499–1515. 10.1111/pce.12266 24393025

[B14] Darienko T. Gustavs L. Eggert A. Wolf W. Pröschold T. (2015). Evaluating the species boundaries of green microalgae (*Coccomyxa*, Trebouxiophyceae, Chlorophyta) using integrative taxonomy and DNA barcoding with further implications for the species identification in environmental samples. *PLoS One* 10:e0127838. 10.1371/journal.pone.0127838 26080086 PMC4469705

[B15] Darienko T. Gustavs L. Mudimu O. Menendez C. R. Schumann R. Karsten U. (2010). *Chloroidium*, a common terrestrial coccoid green alga previously assigned to *Chlorella* (Trebouxiophyceae, Chlorophyta). *Eur. J. Phycol.* 45 79–95. 10.1080/09670260903362820

[B16] Darienko T. Gustavs L. Pröschold T. (2016). Species concept and nomenclatural changes within the genera *Elliptochloris* and *Pseudochlorella* (Trebouxiophyceae) based on an integrative approach. *J. Phycol.* 52 1125–1145. 10.1111/jpy.12481 27734501

[B17] Darienko T. Rad-Menendez C. Campbell C. Pröschold T. (2019). Are there any true marine *Chlorella* species? Molecular phylogenetic assessment and ecology of marine *Chlorella*-like organisms, including a description of *Droopiella* gen. nov. *Syst. Biodivers.* 17 811–829. 10.1080/14772000.2019.1690597 32256217 PMC7077435

[B18] de-Bashan L. E. Trejo A. Huss V. A. Hernandez J. P. Bashan Y. (2008). *Chlorella sorokiniana* UTEX 2805, a heat and intense, sunlight-tolerant microalga with potential for removing ammonium from wastewater. *Bioresour. Technol.* 99 4980–4989. 10.1016/j.biortech.2007.09.065 18024023

[B19] Donner A. Glaser K. Borchhardt N. Karsten U. (2017). Ecophysiological response on dehydration and temperature in terrestrial *Klebsormidium* (Streptophyta) isolated from biological soil crusts in Central European grasslands and forests. *Microb. Ecol.* 73 850–864. 10.1007/s00248-016-0917-3 28011994

[B20] Farrant J. M. Moore J. P. (2011). Programming desiccation-tolerance: from plants to seeds to resurrection plants. *Curr. Opin. Plant Biol.* 14 340–345. 10.1016/j.pbi.2011.03.018 21511516

[B21] Fiehn O. (2016). Metabolomics by gas chromatography-mass spectrometry: combined targeted and untargeted profiling. *Curr. Protoc. Mol. Biol.* 114 30.4.1–30.4.32. 27038389 10.1002/0471142727.mb3004s114 PMC4829120

[B22] Foyer C. H. Noctor G. (2000). Oxygen processing in photosynthesis: regulation and signalling. *New Phytol.* 146 359–388. 10.1046/j.1469-8137.2000.00667.x

[B23] Fuell C. Elliott K. A. Hanfrey C. C. Franceschetti M. Michael A. J. (2010). Polyamine biosynthetic diversity in plants and algae. *Plant Physiol. Biochem.* 48 513–520. 10.1016/j.plaphy.2010.02.008 20227886

[B24] Galili G. Amir R. Fernie A. R. (2016). The regulation of essential amino acid synthesis and accumulation in plants. *Annu. Rev. Plant Biol.* 67 153–178. 10.1146/annurev-arplant-043015-112213 26735064

[B25] Gasulla F. Vom Dorp K. Dombrink I. Zahringer U. Gisch N. Dormann P. Bartels D. (2013). The role of lipid metabolism in the acquisition of desiccation tolerance in *Craterostigma plantagineum*: a comparative approach. *Plant J.* 75 726–741. 10.1111/tpj.12241 23672245

[B26] Gasulla F. Barreno E. Parages M. L. Cámara J. Jiménez C. Dörmann P. (2016). The role of phospholipase D and MAPK signaling cascades in the adaption of lichen microalgae to desiccation: changes in membrane lipids and phosphoproteome. *Plant Cell Physiol.* 57 1908–1920. 10.1093/pcp/pcw111 27335354

[B27] Gerna D. Roach T. Arc E. Stöggl W. M. Limonta M. Vaccino P. (2018). Redox poise and metabolite changes in bread wheat seeds are advanced by priming with hot steam. *Biochem. J.* 475 3725–3743. 10.1042/bcj20180632 30401685

[B28] Glaser K. Donner A. Albrecht M. Mikhailyuk T. Karsten U. (2017). Habitat-specific composition of morphotypes with low genetic diversity in the green algal genus *Klebsormidium* (Streptophyta) isolated from biological soil crusts in Central European grasslands and forests. *Eur. J. Phycol.* 52 188–199. 10.1080/09670262.2016.1235730

[B29] Gray D. W. Lewis L. A. Cardon Z. G. (2007). Photosynthetic recovery following desiccation of desert green algae (Chlorophyta) and their aquatic relatives. *Plant Cell Environ.* 30 1240–1255. 10.1111/j.1365-3040.2007.01704.x 17727415

[B30] Gustavs L. Eggert A. Michalik D. Karsten U. (2010). Physiological and biochemical responses of green microalgae from different habitats to osmotic and matric stress. *Protoplasma* 243 3–14. 10.1007/s00709-009-0060-9 19585217

[B31] Gustavs L. Görs M. Karsten U. (2011). Polyol patterns in biofilm-forming aeroterrestrial green algae (Trebouxiophyceae, Chlorophyta). *J. Phycol.* 47 533–537. 10.1111/j.1529-8817.2011.00979.x 27021982

[B32] Gustavs L. Schumann R. Eggert A. Karsten U. (2009). *In vivo* growth fluorometry: accuracy and limits of microalgal growth rate measurements in ecophysiological investigations. *Aquat. Microb. Ecol.* 55 95–104. 10.3354/ame01291

[B33] Hayat S. Hayat Q. Alyemeni M. N. Wani A. S. Pichtel J. Ahmad A. (2012). Role of proline under changing environments. A review. *Plant Signal. Behav.* 7 1456–1466. 10.4161/psb.21949 22951402 PMC3548871

[B34] Herburger K. Lewis L. A. Holzinger A. (2015). Photosynthetic efficiency, desiccation tolerance and ultrastructure in two phylogenetically distinct strains of alpine *Zygnema* sp. (Zygnematophyceae, Streptophyta): role of pre-akinete formation. *Protoplasma* 252 571–589. 10.1007/s00709-014-0703-3 25269628 PMC4335129

[B35] Hodač L. Hallmann C. Spitzer K. Elster J. Fasshauer F. Brinkmann N. (2016). Widespread green algae *Chlorella* and *Stichococcus* exhibit polar-temperate and tropical-temperate biogeography. *FEMS Microbiol. Ecol.* 92:fiw122. 10.1093/femsec/fiw122 27279416

[B36] Jackson A. E. Seppelt R. D. (1995). The accumulation of proline in *Prasiola crispa* during winter in Antarctica. *Physiol. Plant.* 94 25–30. 10.1034/j.1399-3054.1995.940104.x 11841302

[B37] Kranner I. Birtić S. Anderson K.M. Pritchard H. W. (2006). Glutathione half-cell reduction potential: a universal stress marker and modulator of programmed cell death? *Free Radical Bio. Med.* 40 2155–2165. 10.1016/j.freeradbiomed.2006.02.013 16785029

[B38] Karsten U. Herburger K. Holzinger A. (2014). Dehydration, temperature, and light tolerance in members of the aeroterrestrial green algal genus *Interfilum* (Streptophyta) from biogeographically different temperate soils. *J. Phycol.* 50 804–816. 10.1111/jpy.12210 25810561 PMC4370238

[B39] Karsten U. Holzinger A. (2012). Light, temperature, and desiccation effects on photosynthetic activity, and drought-induced ultrastructural changes in the green alga *Klebsormidium dissectum* (Streptophyta) from a terrestrial soil crust. *Microb. Ecol.* 63 51–63. 10.1007/s00248-011-9924-6 21811791

[B40] Karsten U. Holzinger A. (2014). Green algae in alpine biological soil crust communities: acclimation strategies against ultraviolet radiation and dehydration. *Biodivers. Conserv.* 23 1845–1858. 10.1007/s10531-014-0653-2 24954980 PMC4058318

[B41] Kind T. Wohlgemuth G. Lee D. Y. Lu Y. Palazoglu M. Shahbaz S. (2009). FiehnLib: mass spectral and retention index libraries for metabolomics based on quadrupole and time-of-flight gas chromatography/mass spectrometry. *Anal. Chem.* 81 10038–10048. 10.1021/ac9019522 19928838 PMC2805091

[B42] Kopka J. Schauer N. Krueger S. Birkemeyer C. Usadel B. Bergmüller E. (2005). GMD@CSB.DB: the Golm Metabolome Database. *Bioinformatics* 21 1635–1638. 10.1093/bioinformatics/bti236 15613389

[B43] Körner C. Paulsen J. Pelaez-Riedl S. (2003). “A bioclimatic characterisation of Europe’s alpine areas,” in *Alpine Biodiversity in Europe. Ecological Studies (Analysis and Synthesis)*, eds Nagy L. Grabherr G. Körner C. Thompson D. B. A. (Heidelberg: Springer), 13–28. 10.1007/978-3-642-18967-8_2

[B44] Kosugi M. Miyake H. Yamakawa H. Shibata Y. Miyazawa A. Sugimura T. (2013). Arabitol provided by lichenous fungi enhances ability to dissipate excess light energy in a symbiotic green alga under desiccation. *Plant Cell Physiol.* 54 1316–1325. 10.1093/pcp/pct079 23737501

[B45] Kranner I. Beckett R. Hochman A. Nash T. H. (2008). Desiccation-tolerance in lichens: a review. *Bryologist* 111 576–593. 10.1639/0007-2745-111.4.576

[B46] Kranner I. Birtic S. (2005). A modulating role for antioxidants in desiccation tolerance. *Integr. Comp. Biol.* 45 734–740. 10.1093/icb/45.5.734 21676824

[B47] Kranner I. Cram W. J. Zorn M. Wornik S. Yoshimura I. Stabentheiner E. (2005). Antioxidants and photoprotection in a lichen as compared with its isolated symbiotic partners. *Proc. Natl. Acad. Sci. U.S.A.* 102 3141–3146. 10.1073/pnas.0407716102 15710882 PMC549463

[B48] Lakeman M. B. von Dassow P. Cattolico R. A. (2009). The strain concept in phytoplankton ecology. *Harmful Algae* 8 746–758. 10.1016/j.hal.2008.11.011

[B49] Lenth R. V. (2016). Least-Squares Means: the R Package lsmeans. *J. Stat. Softw.* 69 1–33.

[B50] Luo W. Pröschold T. Bock C. Krienitz L. (2010). Generic concept in *Chlorella*-related coccoid green algae (Chlorophyta, Trebouxiophyceae). *Plant Biol.* 12 545–553. 10.1111/j.1438-8677.2009.00221.x 20522192

[B51] Marin B. Palm A. Klingberg M. Melkonian M. (2003). Phylogeny and taxonomic revision of plastid-containing euglenophytes based on SSU rDNA sequence comparisons and synapomorphic signatures in the SSU rRNA secondary structure. *Protist* 154 99–145. 10.1078/143446103764928521 12812373

[B52] Morita M. Watanabe Y. Saiki H. (2000). High photosynthetic productivity of green microalga *Chlorella sorokiniana*. *Appl. Biochem. Biotechnol.* 87 203–218. 10.1385/abab:87:3:203 10982230

[B53] Müller J. Friedl T. Hepperle D. Lorenz M. Day J. G. (2005). Distinction between multiple isolates of *Chlorella vulgaris* (Chlorophyta, Trebouxiophyceae) and testing for conspecificity using amplified fragment length polymorphism and its rDNA sequences. *J. Phycol.* 41 1236–1247. 10.1111/j.1529-8817.2005.00134.x

[B54] Pérez-Pérez M. E. Couso I. Crespo J. L. (2017). The TOR Signaling Network in the Model Unicellular Green Alga *Chlamydomonas reinhardtii*. *Biomolecules* 7:54. 10.3390/biom7030054 28704927 PMC5618235

[B55] Pfaff S. Borchhardt N. Boy J. Karsten U. Gustavs L. (2016). Desiccation tolerance and growth-temperature requirements of *Coccomyxa* (Trebouxiophyceae, Chlorophyta) strains from Antarctic biological soil crusts. *Algol. Stud.* 151/152 3–19. 10.1127/algol_stud/2016/0245

[B56] Ponnu J. Wahl V. Schmid M. (2011). Trehalose-6-Phosphate: connecting plant metabolism and development. *Front. Plant Sci.* 2:70. 10.3389/fpls.2011.00070 22639606 PMC3355582

[B57] Porra R. J. Thompson W. A. Kriedemann P. E. (1989). Determination of accurate extinction coefficients and simultaneous-equations for assaying chlorophyll-a and chlorophyll-b extracted with 4 different solvents - verification of the concentration of chlorophyll standards by atomic-absorption spectroscopy. *Biochim. Biophys. Acta* 975 384–394. 10.1016/s0005-2728(89)80347-0

[B58] Prasad D. P. V. (1983). Hypoxanthine and allantoin as nitrogen sources for the growth of some freshwater green algae. *New Phytol.* 93 575–580. 10.1111/j.1469-8137.1983.tb02708.x

[B59] Proctor M. C. F. Oliver M. J. Wood A. J. Alpert P. Stark L. R. Cleavitt N. L. (2007). Desiccation-tolerance in bryophytes: a review. *Bryologist* 110 595–621. 10.1639/0007-2745(2007)110[595:DIBAR]2.0.CO;2

[B60] R Core Team (2020). *R: A Language and Environment for statistical Computing.* Vienna: R Foundation for Statistical Computing.

[B61] Remias D. Karsten U. Lütz C. Leya T. (2010). Physiological and morphological processes in the Alpine snow alga *Chloromonas nivalis* (Chlorophyceae) during cyst formation. *Protoplasma* 243 73–86. 10.1007/s00709-010-0123-y 20229328

[B62] Rippin M. Pichrtova M. Arc E. Kranner I. Becker B. Holzinger A. (2019). Metatranscriptomic and metabolite profiling reveals vertical heterogeneity within a *Zygnema* green algal mat from Svalbard (High Arctic). *Environ. Microbiol.* 21 4283–4299. 10.1111/1462-2920.14788 31454446 PMC6899726

[B63] Ronquist F. Huelsenbeck J. P. (2003). MrBayes 3: bayesian phylogenetic inference under mixed models. *Bioinformatics* 19 1572–1574. 10.1093/bioinformatics/btg180 12912839

[B64] Sadowsky A. Mettler-Altmann T. Ott S. (2016). Metabolic response to desiccation stress in strains of green algal photobionts (*Trebouxia*) from two Antarctic lichens of southern habitats. *Phycologia* 55 703–714. 10.2216/15-127.1

[B65] Safi C. Zebib B. Merah O. Pontalier P. Y. Vaca-Garcia C. (2014). Morphology, composition, production, processing and applications of *Chlorella vulgaris*: a review. *Renew. Sustain. Energy Rev.* 35 265–278. 10.1016/j.rser.2014.04.007

[B66] Schreiber U. Bilger W. (1993). Progress in chlorophyll fluorescence research: major developments during the past years in retrospect. *Prog. Bot.* 54 151–173. 10.1007/978-3-642-78020-2_8

[B67] Shukla S. P. Kviderova J. Triska J. Elster J. (2013). *Chlorella mirabilis* as a potential species for biomass production in low-temperature environment. *Front. Microbiol.* 4:97. 10.3389/fmicb.2013.00097 23630521 PMC3632980

[B68] Takagi H. Ishiga Y. Watanabe S. Konishi T. Egusa M. Akiyoshi N. (2016). Allantoin, a stress-related purine metabolite, can activate jasmonate signaling in a MYC2-regulated and abscisic acid-dependent manner. *J. Exp. Bot.* 67 2519–2532. 10.1093/jxb/erw071 26931169 PMC4809300

[B69] Tamura K. Stecher G. Peterson D. Filipski A. Kumar S. (2013). MEGA6: molecular evolutionary genetics analysis version 6.0. *Mol. Biol. Evol.* 30 2725–2729. 10.1093/molbev/mst197 24132122 PMC3840312

[B70] Treves H. Murik O. Kedem I. Eisenstadt D. Meir S. Rogachev I. (2017). Metabolic flexibility underpins growth capabilities of the fastest growing alga. *Curr. Biol.* 27:e2553. 10.1016/j.cub.2017.07.014 28803869

[B71] Vertucci C. W. Farrant J. M. (1995). “Acquisition and loss of desiccation tolerance,” in *Seed Development and Germination*, eds Kigel J. Galili G. (New York, NY: Marcel Dekker), 237–271. 10.1201/9780203740071-10

[B72] Walsby A. E. (1997). Numerical integration of phytoplankton photosynthesis through time and depth in a water column. *New Phytol.* 136 189–209. 10.1046/j.1469-8137.1997.00736.x

[B73] Walters C. Hill L. M. Wheeler J. (2005). Dying while dry: kinetics and mechanisms of deterioration in desiccated organisms. *Integr. Comp. Biol.* 45 751–758. 10.1093/icb/45.5.751 21676826

[B74] Webb W. L. Newton M. Starr D. (1974). Carbon dioxide exchange of *Alnus rubra*: a mathematical model. *Oecologia* 17 281–291. 10.1007/bf00345747 28308943

[B75] Wickham H. (2016). *ggplot2. Elegant Graphics for Data Analysis*, 2nd Edn New York, NY: Springer.

[B76] Winter G. Todd C. D. Trovato M. Forlani G. Funck D. (2015). Physiological implications of arginine metabolism in plants. *Front. Plant Sci.* 6:534. 10.3389/fpls.2015.00534 26284079 PMC4520006

[B77] Zuker M. (2003). Mfold web server for nucleic acid folding and hybridization prediction. *Nucleic Acids Res.* 31 3406–3415. 10.1093/nar/gkg595 12824337 PMC169194

[B78] Zwickl D. J. (2006). *Genetic Algorithm Approaches for the Phylogenetic Analysis of Large Biological Sequence Datasets Under the Maximum Likelihood Criterion.* Ph.D. thesis, The University of Arizona, Tucson, AZ, 125.

