# Supplementary figures and images for: Adaptation to Aquatic and Terrestrial Environments in Chlorella vulgaris (Chlorophyta)
Source: Front Microbiol. 2020 Oct 15;11:585836. doi: 10.3389/fmicb.2020.585836 (PMC7593248; doi:10.3389/fmicb.2020.585836)

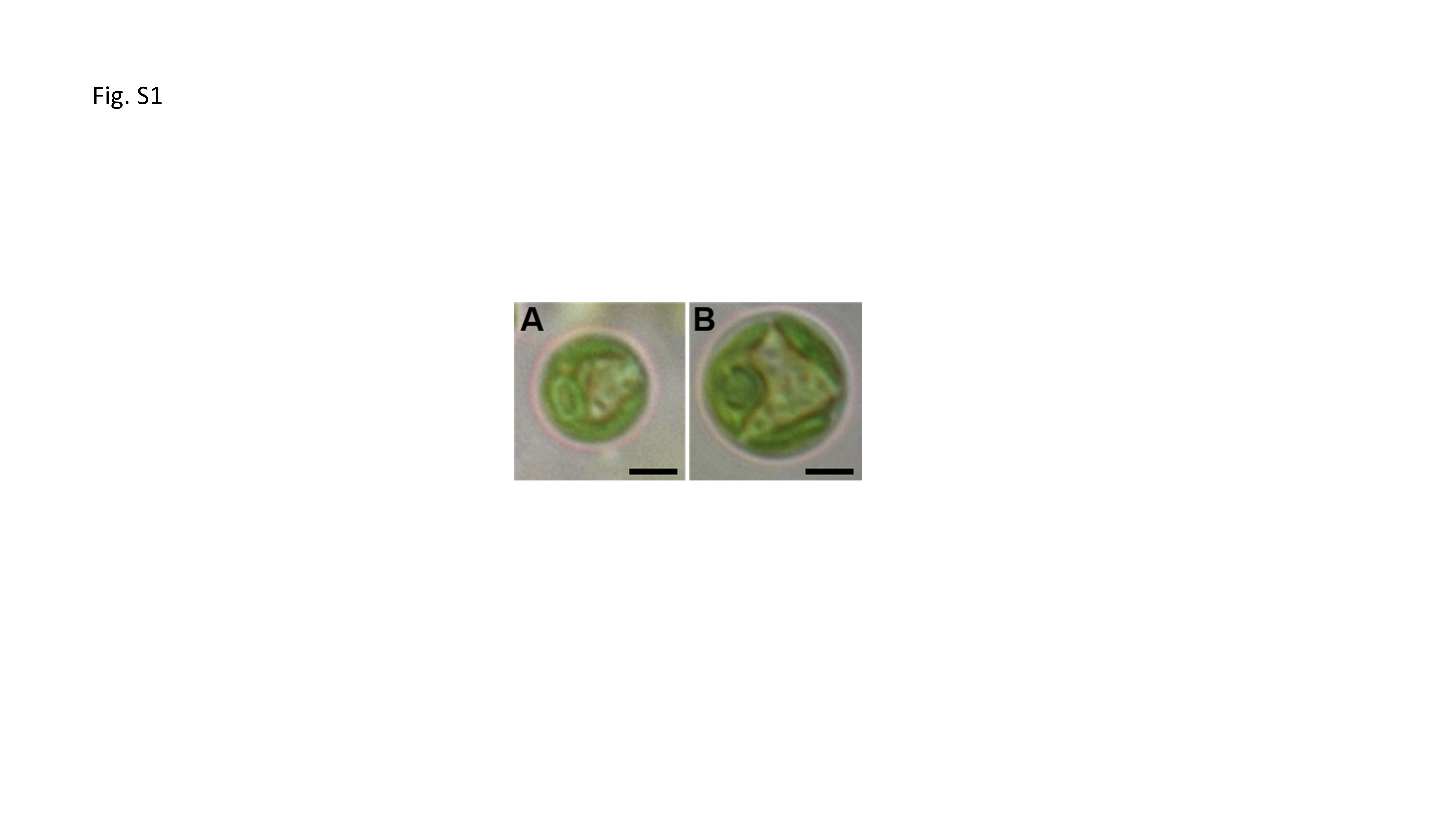

Supplement: Supplementary Figure 1 — Light-micrographs of the two Chlorella vulgaris strains. (A) Representative cell of the authentic aquatic strain (SAG 211-11b) and panel (B) of the terrestrial high alpine strain (ASIB BB67) taken from cultures in the exponential growth phase; scale bars: 2 μm. [file Image_1.TIF]

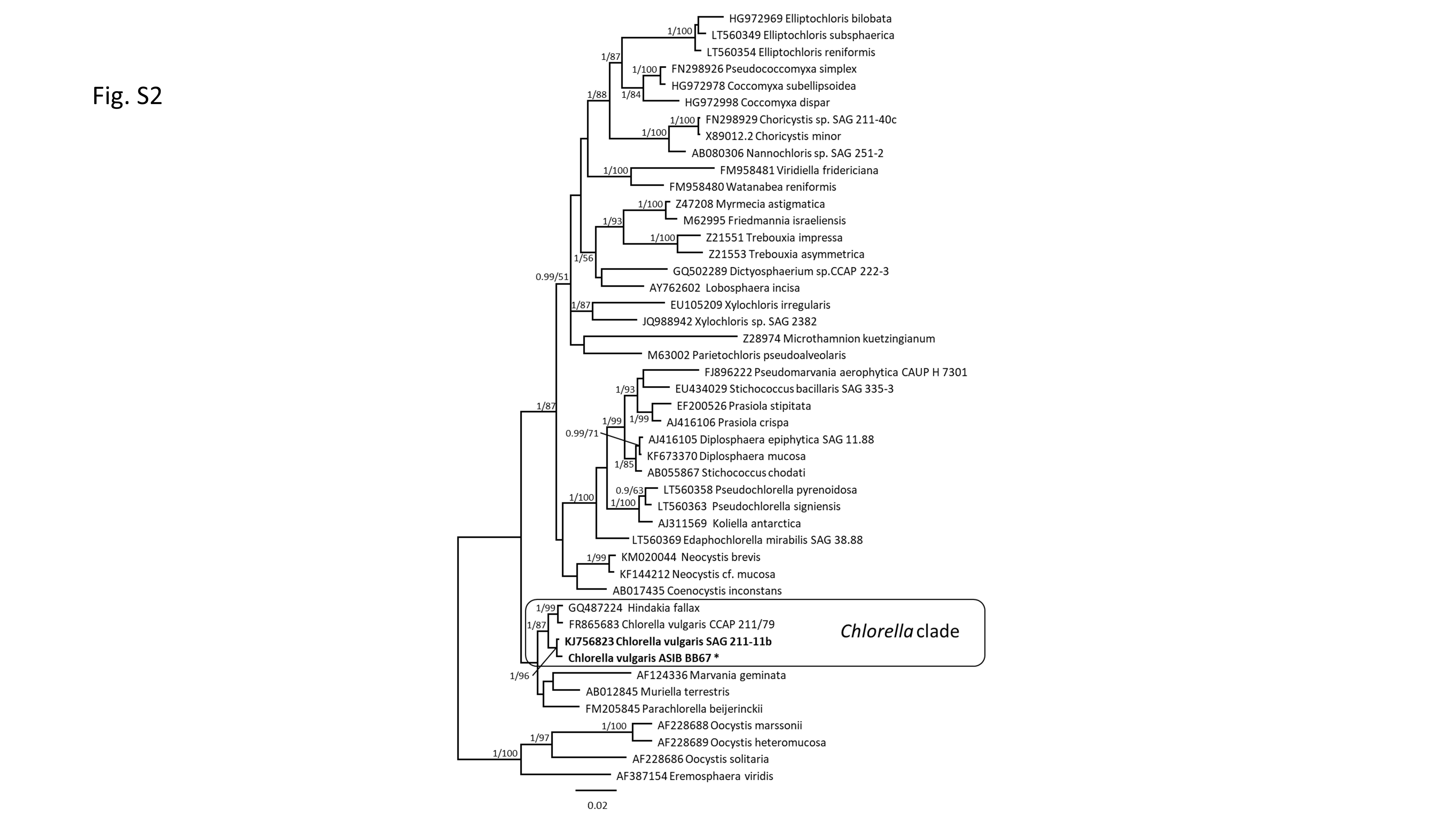

Supplement: Supplementary Figure 2 — Molecular phylogeny of the Trebouxiophyceae. Based on SSU rDNA sequence comparisons, inferred by Bayesian method with Bayesian Posterior Probabilities (PP) and maximum-likelihood (ML) bootstrap support (BP), nodes are indicated based on a data set of 1780 aligned positions of 47 taxa using MrBayes and GARLI. From left to right: support values correspond to ML, BP and Bayesian PP; BP values lower than 50% and PP lower than 0.8 not shown. The sister group of the Oocystis-lineage was chosen as outgroup. The newly sequenced strain BB67 is marked with an asterisk. [file Image_2.TIF]

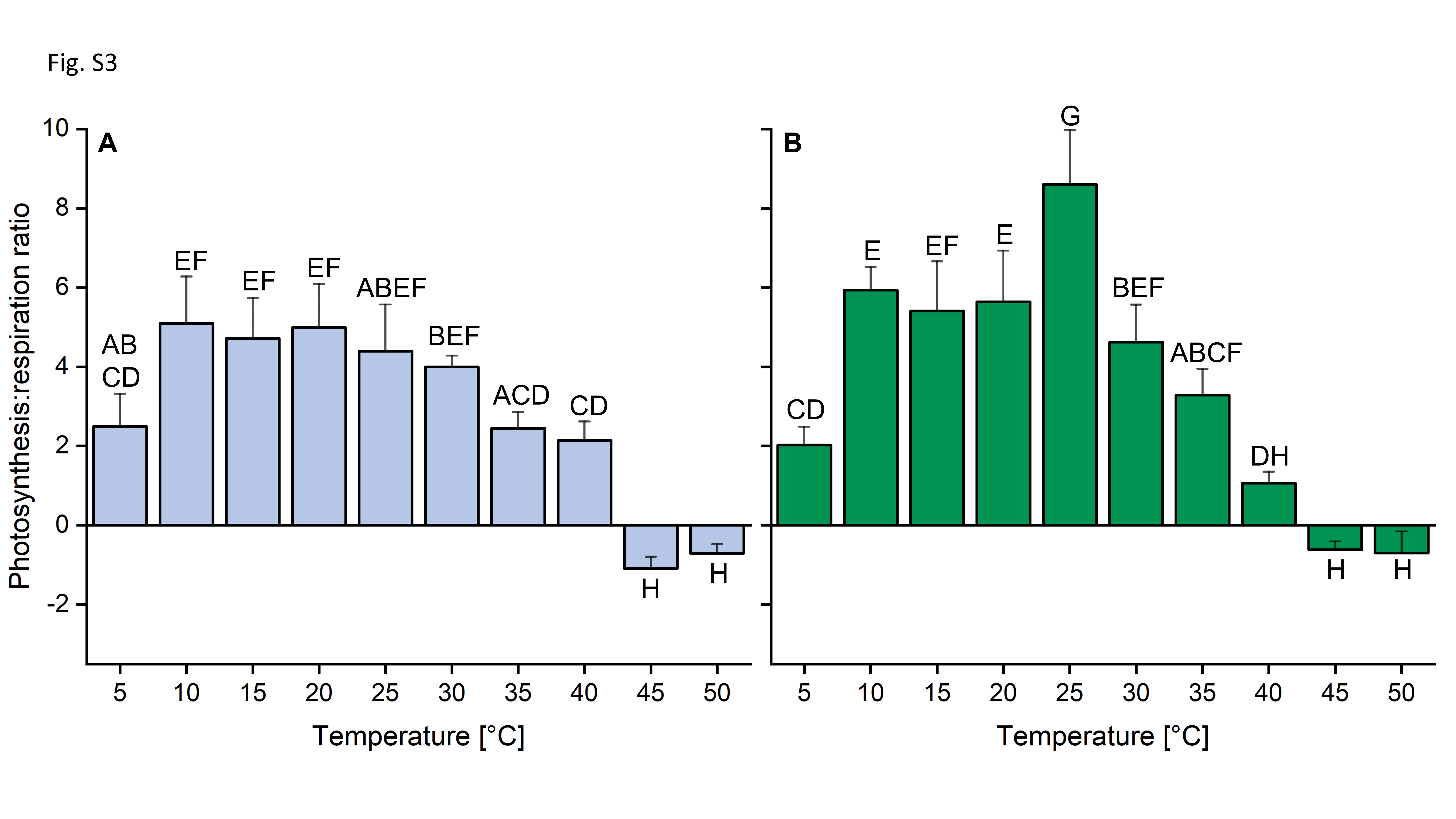

Supplement: Supplementary Figure 3 — Effects of temperature on photosynthesis:respiration (P:R) ratios in two Chlorella vulgaris strains. In panel (A), blue bars show the aquatic strain and in panel (B) green bars show the terrestrial strain; data are means ± SD (n = 4). Capital letters above bars indicate significant differences assessed by two-way ANOVA followed by Tukey’s post hoc test (P < 0.05). [file Image_3.TIF]
