# Supplementary material for: Adaptation to Aquatic and Terrestrial Environments in Chlorella vulgaris (Chlorophyta)
Source: Front Microbiol. 2020 Oct 15;11:585836. doi: 10.3389/fmicb.2020.585836 (PMC7593248; doi:10.3389/fmicb.2020.585836)
Supplement: Supplementary Table 2 — Concentrations of photosynthetic pigments and in two Chlorella vulgaris strains before dehydration (controls); data are means ± SD (n = 3), and values did not differ statistically (P < 0.05). [file Table_2.docx]

**Table S****2.** Concentrations of photosynthetic pigments and in two *Chlorella* *vulgaris* strains before dehydration (controls); data are means ± SD (*n* = 3), and values did not differ statistically (*P* < 0.05).

| Photosynthetic pigments  (nmol mg^-1^ dry weight) | Aquatic  *Chlorella vulgaris* | Terrestrial  *Chlorella vulgaris* |
| --- | --- | --- |
| Chlorophyll a | 32.60 ± 6.31 | 28.4 ± 7.11 |
| Chlorophyll b | 4.91 ± 1.08 | 3.53 ± 1.15 |
| Lutein | 9.02 ± 1.78 | 7.19 ± 1.35 |
| Zeaxanthin | 0.49 ± 0.2 | 0.47 ± 0.25 |
| Violaxanthin | 2.35 ± 0.6 | 1.75 ± 0.67 |
| Antheraxanthin | 0.29 ± 0.15 | 0.24 ± 0.12 |
| Neoxanthin | 1.68 ± 0.29 | 1.38 ± 0.36 |
| β-Carotene | 1.88 ± 0.2 | 1.72 ± 0.42 |
